# Deep Learning in Neuroimaging: Overcoming Challenges With Emerging Approaches

**DOI:** 10.3389/fpsyt.2022.912600

**Published:** 2022-06-02

**Authors:** Jason Smucny, Ge Shi, Ian Davidson

**Affiliations:** ^1^Department of Psychiatry and Behavioral Sciences, University of California, Davis, Davis, CA, United States; ^2^Department of Computer Sciences, University of California, Davis, Davis, CA, United States

**Keywords:** deep learning, mixup data augmentation, transfer learning, explainable AI, fMRI

## Abstract

Deep learning (DL) is of great interest in psychiatry due its potential yet largely untapped ability to utilize multidimensional datasets (such as fMRI data) to predict clinical outcomes. Typical DL methods, however, have strong assumptions, such as large datasets and underlying model opaqueness, that are suitable for natural image prediction problems but not medical imaging. Here we describe three relatively novel DL approaches that may help accelerate its incorporation into mainstream psychiatry research and ultimately bring it into the clinic as a prognostic tool. We first introduce two methods that can reduce the amount of training data required to develop accurate models. These may prove invaluable for fMRI-based DL given the time and monetary expense required to acquire neuroimaging data. These methods are (1) *transfer learning* − the ability of deep learners to incorporate knowledge learned from one data source (e.g., fMRI data from one site) and apply it toward learning from a second data source (e.g., data from another site), and (2) *data augmentation (via Mixup)* − a self-supervised learning technique in which “virtual” instances are created. We then discuss *explainable artificial intelligence* (XAI), i.e., tools that reveal what features (and in what combinations) deep learners use to make decisions. XAI can be used to solve the “black box” criticism common in DL and reveal mechanisms that ultimately produce clinical outcomes. We expect these techniques to greatly enhance the applicability of DL in psychiatric research and help reveal novel mechanisms and potential pathways for therapeutic intervention in mental illness.

## Introduction: Deep Learning and Functional Magnetic Resonance Imaging

The past several years has seen an explosion of interest in machine learning (ML) applications for functional magnetic resonance imaging (fMRI). To illustrate, a PubMed search for “fMRI machine learning” yields a roughly exponential increase in results from 2010 to 2020, with 39 hits in 2010, 300 in 2015, and 1,165 in 2020. Of particular significance is in developing fMRI-compatible ML tools for clinical mental health applications, such as predicting clinical response to treatment. Such forecasting remains a critically unmet challenge as clinical data alone is typically insufficient to predict response. As a result, the process of prescribing ideal treatment regimens often requires clinician adjustment over a substantial period (months to years). Patients with psychosis, for example, may be prescribed various medications until a suitable one is found, increasing the cost and potential risks of treatment (1).

Despite this surge of interest, fMRI-based ML has not yet become a component of standard clinical diagnoses. Indeed, *shallow* ML algorithms, such as support vector machines and random forests, have not yet consistently demonstrated they can predict treatment outcomes with sufficient accuracy to be useful clinical tools. A limitation of these algorithms is that the data features used for prediction must be selected beforehand. Although this may be done with some degree of success using *a priori* hypotheses or data-driven regularization methods such as LASSO regression (2), ideally a ML algorithm would be able to teach itself how to select features as well as combine them in meaningful ways to maximize performance.

Accordingly, end-to-end learning is a prominent feature of more recently developed, deep learning (DL) algorithms. DL algorithms perform feature selection by combining raw data into successively more complex and useful composite representations [(3); see Koppe et al. (4) for a review of DL as applied to neuroimaging data in mental health]. By creating these representations, the deep learner can increase its computational capacity to discover predictive functions with optimum efficiency. In this manner, it may maximize the predictive power provided by its input data, resulting in better performance compared to shallow architectures. Indeed, some evidence suggests that deep learners outperform shallow ML classifiers when using fMRI data (5–7), including recent studies using task fMRI data to predict clinical improvement in recent onset schizophrenia [logistic regression in Smucny et al. (8); shallow ML and DL architectures in Smucny et al. (7)]. Furthermore, a recent meta-analysis comparing DL to shallow ML when using neuroimaging to classify psychiatric disorders (autism, attention deficit hyperactivity disorder, and schizophrenia) found overall qualitatively higher odds ratios when using DL, although the difference was only statistically significant for autism (9).

A classic example of the power of DL is in image prediction and classification, in which specialized DNNs called convolutional neural networks (CNNs) combine line features to form more and more complex shapes and ultimately objects (10). CNNs are particularly effective at preventing overfitting as, due to weight sharing, the number of weights available for training is reduced. CNNs can thus extract local patterns independent of precise locations and find progressively complex patterns with layer depth. In functional neuroimaging contexts such patterns may be represented by increasingly complex patterns of spatial activation (11), blood oxygen-dependent response functions (if time series are used) (11, 12), or network connectivity (13). Feature selection and convolution are ascertained as part of the DL process. Although they are feed-forward in nature, CNNs also incorporate a backpropagation algorithm during training to perform adjustments to internal parameters that are used to compute the representation in each layer from the representation in the previous layer (14, 15).

A second class of DNNs called recurrent neural networks (RNNs) may be particularly applicable in functional neuroimaging as these networks were developed for use in time series data. Akin to autoregressive models in linear regression, RNNs employ previous knowledge of function outputs toward future prediction. These networks may also move back and forth (hence the term “recurrent”), similar to how the brain uses stored knowledge to influence perception while also using perception to update stored knowledge [reviewed by Koppe et al. (4) and Durstewitz et al. (16)].

Despite the advantage of DL over shallow ML architectures, several issues remain in DL (as well as ML in general) which have been problematic when using these architectures to perform neuroimaging data-based classification (17). First, performing feature selection in the face of high dimensional data such as fMRI is a challenge, even for many DL architectures. Second, many deep learners require very large sample sizes to both perform well in a single dataset and generalize to independent datasets. Given the time and monetary expense required to perform neuroimaging studies, such sample sizes may be too large to be feasibly collected without enlisting large consortia. Third, DL has been described as a “black box,” providing predictions without any corresponding output as to what features or feature combinations were used to make decisions. Black-box approaches thus are unable to discern the neuronal mechanisms that underlie the pathology of the disorder to develop targetable biomarkers.

To that end, the purpose of this review is to introduce several extensions of standard DL pipelines that may be used to overcome these challenges. Specifically, we introduce ***transfer learning*** as a method to overcome the high dimensionality challenge and small sample size challenge, ***data augmentation (via Mixup)*** to overcome the small sample size challenge, and ***explainable artificial intelligence (XAI)*** to overcome the opaqueness (“black box”) challenge (Table 1). As they are relatively novel, these DL extensions have yet to be widely utilized in neuroimaging-based psychiatry research, and we hope they may eventually help bring fMRI into the clinic as part of a diagnostic, predictive battery.

**TABLE 1:** Challenges for deep learning on fMRI data and proposed, emerging solutions.

	High dimensional data	Small sample sizes	Opaque interpretability
Transfer learning	X	X	
Data augmentation: mixup		X	
Explainable artificial intelligence			X

### Transfer Learning

**FIGURE 1:**
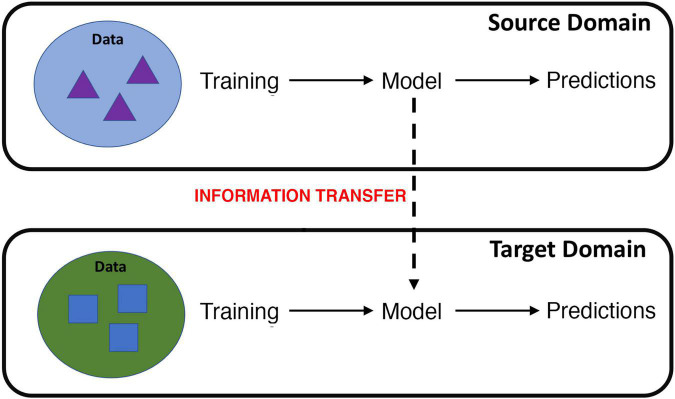
Cartoon illustration of transfer learning, where information learned from a source domain is transferred to learning in a target domain. For example, information learned from fMRI data collected during a particular cognitive task or scanning procedure can be transferred to improve learning on data gathered from a different cognitive task or scanning procedure.

Formally defined by Bozinovski and Fulgosi (18) and first carried out in machine learning by Pratt (19), ***transfer learning*** focuses on applying the knowledge gained while solving one problem to a different but related problem. The definition of transfer learning is illustrated in terms of domains and tasks. Specifically, where the knowledge is transferred from is called the *source domain* and where the knowledge is transferred to is called the *target domain* (Figure 1) (20). Transfer learning aims to help improve the learning of the target predictive function of the target task in the target domain using the knowledge in the source domain and source task, especially when there is scarce data in the target domain and ample data in the source domain. Transfer learning may either use a pre-trained network as a feature extractor or fine-tune a pretrained network on target domain data.

One variant of transfer learning, called *domain adaptation* (20), may be particularly well suited for brain imaging. Domain adaptation occurs when the source and target domains have different distributions (although they must share the same feature space) but the underlying prediction task is the same. In the case of MRI/fMRI data, this may occur when knowledge gained from imaging data collected at one imaging site is transferred to another site, or when knowledge from data collected using one scanning protocol is transferred to data from a different scanning protocol. Domain adaptation may be particularly applicable to fMRI data, as fMRI datasets from individual sites are typically small due to the high cost and resources required. Learning across multiple fMRI sites, however, may also be hampered by “batch” effects in which data from different sites may have different probability distributions, e.g., multivoxel mappings of a disease and control group may be different according to site and/or scanning procedure.

Accordingly, researchers have begun developing domain adaptation algorithms for use in ML across multiple MRI datasets. Although the algorithms used in these studies are different, in general the goal is to find a common feature space over which to transfer knowledge learned from a source domain (e.g., scanning site) to the target domain (another site). An early example is a structural MRI (white matter hyperintensity) study by Ghafoorian et al. (21), who found that adapting knowledge from source domain MRI data with voxel size 1.0 × 1.2 × 5.0 mm toward target domain data with voxel size 1.0 × 1.2 × 3.0 mm improved Dice scores (voxel proportion of true positives) by up to ∼50%. Promotion effects (the discrepancy between the Dice scores on the target domain with vs. without transfer learning) grew as the target set size decreased, illustrating the power of adaptation on small samples. Regarding fMRI, using low rank domain adaptation on a 17-site resting state fMRI dataset, Wang et al. (22) achieved 64–75% accuracy (depending on target site) when identifying children with autism spectrum disorder. Notably, their low rank method, which mapped the high-dimensional, multi (seventeen)-site data to a common, low-rank space, performed ∼5–15% better on average compared with four other classification strategies. Other domain adaptation strategies have also been developed, e.g., a shared space algorithm by Yousefnezhad et al. (23) that classified multisite fMRI task data according to the task being performed with > 90% accuracy, and a Side Information Dependence Regularization framework by Zhou et al. (24) that classified multisite fMRI data by task condition with 79% accuracy. Domain adaptation may also be enriched by data harmonization (e.g., outlier removal, data normalization, data standardization (25). Domain adaptation can also still be effective when the modality of source and target domains use different scanning technologies (26); For example, Chen et al. (27) used domain adaptation to improve heart segmentation in which the source domain consisted of MRI images and the target domain of computerized tomography images.

Transfer learning can also be used to transfer information learned by a machine across tasks *via task transfer*. Using task transfer, a machine can use what it has learned from features in one task (e.g., classifying schizophrenia patients from healthy individuals using imaging data) toward improving classification in another task (e.g., classifying people with bipolar disorder from unaffected people from imaging data). This technique can also enable the machine to learn from imaging data collected from one cognitive task to enhance classification using data from another cognitive task. Perhaps the earliest example of task transfer applied to fMRI data was by Mensch et al. (28) who demonstrated that transferring knowledge from Human Connectome Project (HCP) task data improved accuracy by 1.1–1.6% when predicting cognitive state (e.g., watching faces vs. houses) from fMRI data from other datasets that examined related cognitive tasks. Mirroring the domain adaptation study by Ghafoorian et al. (21) (see previous paragraph), this effect was magnified substantially when target domain sample sizes were small. Thomas et al. (29) also demonstrated the utility of task transfer in a study incorporating a unique DL framework called DeepLight. Specifically, Thomas et al. (29) transferred information used to decode cognitive states from 6 cognitive tasks from the HCP toward deciphering the cognitive state from a 7th task (working memory). The authors demonstrated enhanced performance of the trained learner vs. the naive learner, as the trained, transferred learner only required 40% of the training data sample from the working memory task to achieve significantly higher accuracy on test data vs. a naive learner that used 100% of the training data. Task transfer may also have applicability to fMRI datasets as individuals are frequently asked to perform multiple tasks during a scanning session.

### Data Augmentation by Mixup: An Example of Self-Supervised Learning

Although transfer learning is a powerful technique, it is limited in that while it can transfer the convolutional filters used to identify features, it cannot easily transfer feed forward layers that perform the logical operations to reason about them. A potential issue with transfer learning as applied to neuroimaging data between different sites, furthermore, is that different scanners have different signal/noise ratios and measurement artifacts. In addition to transfer learning, another set of DL methods that can help solve the small data problem typical of neuroimaging analysis are data augmentation methods (which are often considered a form of self-supervised learning). Although there are many of these methods (e.g., affine transformations, padding, and random cropping) (30), due to its potential in neuroimaging analysis we focus here on a relatively recently developed method called *data Mixup*. Mixup is a type of self-supervised learning where the learner self-generates virtually labeled instances as a combination of individual data points (e.g., fMRI activation maps from two individuals) (31). Importantly, these instances help smooth decision boundaries and thereby help prevent overfitting, i.e., the poor generalization of trained models. Indeed, overfitting is a major concern in fMRI analysis as brain signals of interest may be highly influenced by noise [e.g., participant motion (32)]. This overfitting may be especially problematic when sample sizes are small.

*Mixup* creates new virtual instances in a simple yet powerful method by randomly choosing two instances to produce a third that is the weighted average of the two training samples and their labels (31):


$$\underline{n\,e\,w\,d\,a\,t\,a}=\lambda *d\,a\,t\,a\,1+\left(1-\lambda \right)\,d\,a\,t\,a\,2,$$

where λ is a % fraction taken randomly from a beta distribution


$$\underline{n\,e\,w\,l\,a\,b\,e\,l}=\lambda *l\,a\,b\,e\,l\,1+\left(1-\lambda \right)\,l\,a\,b\,e\,l\,2,$$

where λ is a % fraction taken randomly from a beta distribution

In these equations, data1 and data2 as well as corresponding label1 and label2 are two examples drawn at random from training data.

**FIGURE 2:**
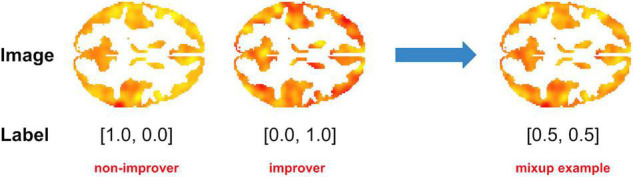
Example of Mixup as applied to fMRI data. In this example, a 50/50 virtual instance was created by combining task (cognitive control-associated) fMRI data from a recent onset patient with schizophrenia with a good clinical outcome [> 20% Improvement in Total Brief Psychiatric Rating score after 1 year of treatment (“Improver”)] with that of a patient with a poor clinical outcome (“Non-Improver”). Data were taken from a study by Smucny et al. (7).

In the context of fMRI data, Mixup may involve combining single-subject imaging data from a person with a particular outcome with data from a person with a different outcome to create a virtual instance (Figure 2). Ratios of additional virtual instances are typically added from a beta distribution (31). By adding these virtual instances into the training samples, the model is given many more variations of existing data. As Mixup smooths out the underlying distribution, it has been shown to aid in regularization, thereby reducing the influence of outliers and consequent overfitting or sensitivity to label corruption/adversarial attacks (33, 34). Notably, although a linear combination of input features and annotated labels is presented, it doesn’t impose a requirement that the learned model’s decision boundary on the input space between classes must be a linear combination of the mixed examples.

To our knowledge, Mixup has not yet been utilized in fMRI-based DL architectures, although it has been used to improve structural MRI image classification [e.g., segment knee cartilage (35) or classify brain gliomas (36)]. Regarding whole-brain imaging data, Bron et al. (37) compared performance between a support vector machine (SVM) and CNN with Mixup-augmented samples on using structural MRI data to (1) classify patients with Alzheimer’s Disease (AD) vs. controls, and (2) classify people with mild cognitive impairment into future AD converters or non-converters. Bron et al. (37) also compared performance using minimally preprocessed and heavily preprocessed T1 maps, as well as between the same vs. an independent dataset. The investigators found that Mixup-augmented CNNs performed qualitatively better, with ∼3–5% accuracy improvement, than SVMs on the independent dataset for these tasks. The SVM, however, was better at predicting conversion on the dataset from which training samples were obtained. It should also be noted that Bron et al. (37) used a static Mixup fraction (0.8/0.2) as opposed to the recommended range of fractions derived from the beta distribution, potentially reducing the predictive power of their CNN. Furthermore, given that they are computationally different algorithms, CNNs may not be directly comparable to SVMs.

Despite its demonstrated effectiveness, the power of Mixup as initially formulated may be limited because the synthetic samples it generates exist on the linear interpolation space of in-distribution samples (38). Thus, for a pair of random samples in 2D training set, synthetic samples are created on the straight line between the pair. Manifold intrusion may also occur if a mixed example collides with a real example in the data manifold, but is given a soft label that is different from the label of the real example leading to underfitting (39). To address these issues, a more complex Mixup method called non-linear Mixup was recently introduced (38). In non-linear Mixup, synthetic samples are independently created on each dimension on the input, adding an additional dimension to the synthetic space. Furthermore, non-linear Mixup enables labels to be adaptively learned based on the synthetic input, reducing the likelihood of manifold intrusion. Other recent methodologic enhancements to “standard” Mixup include manifold Mixup, in which intermediate layers of neural networks are mixed to generate more realistic instances (40), and XMixup (41), in which Mixup is combined with transfer learning by mixing up examples across different domains.

### Explainable Artificial Intelligence

Deep learning models are extremely complex and opaque to humans and are therefore often criticized for being a “black box.” To shed this image, it is essential for deep learners to not only output performance metrics but also the information discovered to be most essential to prediction as part of the deep learning process. The Defense Advanced Research Projects Agency has further delineated the necessity of XAI in DL (42). In general, XAI is designed to address issues (43) such as: (1) Why did you predict that? (2) Does your rule make sense? and (3) Can I trust you?

The goals of XAI are thus not only to enhance transparency, but also enable a domain expert to examine the learned features, understand the decision-making process of the model, find the drawbacks of existing design, improve the design, and, if necessary, reconstruct the learner. In the context of neuroimaging, XAI outputs may include brain regions or connectivity patterns that most influence prediction. One may thus imagine that such output is required to not only enhance machine trustworthiness (are the brain regions important for prediction consistent with those predicted by theory?) but also biomarker development (can we design interventions that specifically target those regions to improve outcomes?). One may further imagine that XAI outputs may be used to identify biologically defined subgroups of individuals that may be agnostic to primary psychiatric diagnosis, consistent with frameworks such as the Research Domain Criteria (44, 45). Indeed, the NIH has recognized the importance of XAI in human neuroscience research with an R01 funding opportunity.^1^ Examples of XAI techniques that may be useful in when performing DL on fMRI data include the following:

#### Saliency Mapping Methods

These methods provide individual instance level explanations. In the context of brain imaging, they output individual heat maps, similar to statistical parametric maps outputted in voxelwise fMRI analysis, that illustrate the importance of particular voxels in their contribution to the decision for that scan (46). The values are combined to form a map for each unique input example that corresponds to discriminative features in the input space for classifications.

#### Signal Reconstruction Methods

These methods output feature level explanations, such as lines, shapes, and higher-level features found in intermediate layers of DL architectures (14). In brain imaging contexts, these may be patterns of activity during task conditions. These styles of explanations are useful for a collection of instances such as correctly predicted instances of a given class.

#### Rule Discovery Methods

Rule discovery methods are examples of model level explanations that extract underlying logical statements (logical rules) that are naturally interpretable by humans. An example of a logical statement is a conditional statement, e.g., if A then B. An illustrative example of rule discovery was recently published by our group in a study using fMRI data to predict clinical improvement in recent onset psychosis (7). Specifically, we found that a deep learner could use fMRI data from 4 frontoparietal ROIs during a cognitive control task to predict clinical improvement after 1 year with 70% accuracy, with the most predictive rule being a baseline level of cognitive control-associated activity in the left dorsolateral prefrontal cortex between the average activation of the patient and unaffected control groups.

#### Increasing Explainability

Notably, the 3 methods above make no guarantee that the XAI output is easily explainable to humans. Indeed, they are post-processing methods which attempt to explain a model rather than generating an explainable-by design model. This may be particularly problematic for rule discovering methods as applied to high dimensional datasets such as fMRI. An emerging area of XAI research is the development of algorithms and strategies that attempt to increase the simplicity of explanations while maintaining a high level of performance. For fMRI, these may be as simple as performing data reduction (e.g., *via* principal component analysis) prior to performing DL with XAI, although may also cause the model to miss important subtleties in the data [reviewed by Yang et al. (47)]. Other methods, such as SINDy regularization (48), impose penalties on model complexity. Rule interpretability and simplicity can also be increased in other ways. One is *via* anchor rules, i.e., if-then rules that predict outcome regardless of other predictors (49). This strategy has been shown to increase understanding and trust in XAI outputs in humans while preserving performance accuracy (49). Other XAI methods are particularly useful for imaging data in that they provide voxel-level explanations, such as (1) model-agnostic explanations (LIME), which test the effects of local perturbations of the data to find which combinations of features are most influential (50), and (2) Shapley Additive exPlanations (SHAP), which use a special weighted linear regression to estimate the importance of each feature (47, 51, 52).

#### Increasing Explainability: Human-in-the-Loop

A common criticism of deep learning (and machine learning in general) is that it is entirely data-driven, ignoring the wisdom and expertise of decades of hypothesis-driven research. One emerging approach is to use expert human knowledge in combination with XAI to develop an interpretable, accuracy model that is consistent with theory (45, 53). In a DL framework, this may involve performing XAI on a learner with a complete feature set, have the DL model generate results consistent with domain expertise, and have the DL re-perform its calculations excluding the rules judged by the expert to be superfluous. A theoretical example in the context of task fMRI would be to focus on rules that involve brain regions known to be associated with the cognitive process of interest. This approach is inherently challenging because it requires a human and machine to speak in a common language and is therefore a largely unexplored area of research, particularly in computational psychiatry and neuroimaging.

## Conclusion

The recent explosion in the application of DL to medical imaging has yielded many promising results. If machines are to augment or even replace humans in diagnosis and prognosis in a variety of situations, however, then several outstanding challenges still need to be addressed. These include the need to choose features, the need to overcome a lack of training data, and the need for explanation. We discuss three techniques to address these challenges recently used in ML but only just beginning to be used in medical imaging: (1) transfer learning, (2) data augmentation, and (3) XAI. We believe a wider appreciation and exploration of these methods will move medical imaging and computational psychiatry forward toward its ideals.

## Data Availability Statement

The original contributions presented in the study are included in the article/supplementary material, further inquiries can be directed to the corresponding author/s.

## Author Contributions

JS, GS, and ID conceived of manuscript concepts and wrote the manuscript. All authors contributed to the article and approved the submitted version.

## Conflict of Interest

The authors declare that the research was conducted in the absence of any commercial or financial relationships that could be construed as a potential conflict of interest.

## Publisher’s Note

All claims expressed in this article are solely those of the authors and do not necessarily represent those of their affiliated organizations, or those of the publisher, the editors and the reviewers. Any product that may be evaluated in this article, or claim that may be made by its manufacturer, is not guaranteed or endorsed by the publisher.

## Footnotes

1
grants.nih.gov/grants/guide/pa-files/PAR-19-344.html
